# Developing a Safety Planning Smartphone App to Support Adolescents’ Self-Management During Emotional Crises

**DOI:** 10.3390/ijerph22111607

**Published:** 2025-10-22

**Authors:** Tamara Großmann, Jana Hörger, Nadine Bayer, Sophie Bückle, Daniel Buschek, Jörg M. Fegert, Peter Laurenz, Matthias Lühr, Franziska Marek, Miriam Rassenhofer, Nathalie Oexle

**Affiliations:** 1 Department of Psychiatry and Psychotherapy II, University of Ulm and BKH Günzburg, 89073 Ulm, Germany; 2 German Center for Mental Health (DZPG), Partner Site Ulm, 89073 Ulm, Germany; 3 Clinic for Child and Adolescent Psychiatry, Psychosomatics and Psychotherapy, Ulm University Medical Center, 89073 Ulm, Germany; 4 Department of Computer Science, University of Bayreuth, 95447 Bayreuth, Germany

**Keywords:** suicidal ideation, adolescent, suicide prevention, safety planning, adolescent psychiatry, self-management, mobile applications, mental health

## Abstract

Suicide is a leading cause of death among adolescents, highlighting the need for effective suicide prevention strategies. Safety planning is a best-practice intervention that has recently shifted toward smartphone-based formats. This study explored stakeholder perspectives (adolescents, parents, practitioners) and described the development of an age-tailored app. A qualitative study was conducted in Germany (2023–2024) with focus groups involving adolescents (*n* = 7), parents (*n* = 4), and practitioners (*n* = 4). Adolescents (14–21 years) were eligible if they had received inpatient treatment, experienced suicidal thoughts within the past 24 months, and had prior experience with safety planning. Parents and practitioners had experience or expertise with suicidality among adolescents. Data were analyzed using Kuckartz’s qualitative content analysis. App development was based, among other things, on insights from focus groups and pertinent theories. Stakeholders expressed differing needs regarding app content, settings, and adjustability. The developed emira-app includes an interactive safety plan to support users in self-managing emotional crises, along with additional features (e.g., digital HopeBox and diary) to promote integration into users’ daily routines. This multi-component safety planning app was specifically developed for adolescents, and its participatory development process allowed an intensive exploration of key stakeholders’ perspectives.

## 1. Introduction

Every year, approximately 700,000 people of all ages die by suicide worldwide [1], highlighting its significance as a public health issue across all age groups. Suicidal thoughts and behaviors are also common among adolescents [2,3,4]. In Germany, 486 people under the age of 25 died by suicide in 2023 [5], underscoring the relevance of suicide as one of the leading causes of death among young people worldwide [1]. Studies conducted in schools further highlight this issue, reporting that between 6.5% and 9% of adolescents have attempted suicide at least once, while the 12-month prevalence of suicidal thoughts is around 14% among 15-year-olds [6]. These prevalence rates are particularly high among adolescents with psychiatric disorders, who are at significantly increased risk for suicidal thoughts and behaviors [7]. Furthermore, such thoughts and behaviours are often persistent or recurrent during adolescence [8], emphasizing the urgent need for effective suicide prevention among this group.

One evidence-based intervention designed to ensure an individual’s safety during a suicidal crisis is safety planning. It provides a structured framework with six safety plan components (SPCs): (1) identifying warning signs, (2) implementing self-management strategies, (3) using social distraction, (4) building a support network of trusted individuals as well as (5) professionals, and (6) eliminating access to suicide methods [9]. Usually, safety plans are developed collaboratively with practitioners, though individuals may also create them independently. The effectiveness of this intervention has been empirically validated among adults. For instance, Stanley et al. [10] demonstrated that safety planning was associated with reduced suicidal behavior and increased treatment engagement among suicidal adults within six months after discharge from the emergency department. These findings are supported by evidence from a recent meta-analysis, demonstrating that safety planning interventions are associated with reduced suicidal behavior, but showed no significant effect on suicidal ideation (SI; [11]). In contrast, Ferguson et al. [12] conducted a systematic review of 26 studies and reported that safety planning interventions may reduce both suicidal behavior and ideation, as well as symptoms of depression and hopelessness. In their systematic review of safety planning interventions for children and adolescents, Abbott-Smith et al. [13] concluded that although current empirical evidence suggests the general applicability of safety planning for use with children and adolescents, more research is needed to assess its effectiveness among this group. Interestingly, May et al. [14] investigated safety plan usage among psychiatrically hospitalized adolescents and found that, while more than 90% accessed their safety plan within the first month post-discharge, safety plan usage sharply declined in the following weeks.

To date, safety plans are usually handwritten on paper [15], potentially limiting their availability and self-application in emotionally stressful situations [16]. Initial studies suggest that some of these limitations could be adequately addressed by smartphone-based safety planning, providing users with the advantage of quick access to the safety plan when needed. In fact, nearly all adolescents own a smartphone. In 2022, 96% of 12- to 19-year-olds in Germany owned one [17]. One study conducted in 2017 suggested that 71% of adolescents about to be discharged from a psychiatric hospital either downloaded a safety planning application (app) or were interested in downloading one, highlighting adolescents’ general interest in using apps—particularly for managing their mental health [18]. Supporting this, Myin-Germeys et al. [19] emphasized their relevance particularly in the context of psychiatric topics, such as the management of severe mental illness. Given this growing interest and their potential benefits, the popularity of smartphone apps for addressing different mental health issues is rapidly increasing.

One example of apps used in the mental health context are those that replicate the therapeutic HopeBox intervention [20,21], allowing users to digitally store their personal reasons for living—such as photos of loved ones—making them easily accessible on a smartphone. Denneson et al. [21] demonstrated that using a digital HopeBox can enhance coping self-efficacy, which may help to reduce SI. Other available apps focus on mood tracking, enabling users to record their emotions and monitor mood patterns over time. According to Dubad et al. [22], mood-tracking apps can be a valuable supplement for adolescents with mental health issues.

Internationally, several apps provide smartphone-based safety planning [15,23,24,25,26,27,28,29]. Some focus exclusively on safety planning, differing in functionality and availability (cf. [30]), while others integrate additional features, such as a digital HopeBox, a diary, or psychoeducation [15,23,26,27,31]. In German-speaking countries, only a few safety planning apps exist (such as SERO [32], KrisenKompass, and LifeStep). Initial research on the effectiveness of safety planning apps indicates their significant contribution to suicide prevention [23,25,26,28,29,33,34]. For example, the safety planning smartphone app BeyondNow has been evaluated in both adult populations [34], and young adults with a mean age of approximately twenty years [25]. Both studies reported a significant reduction in SI, as well as an increase in suicide-related coping—defined as the use of internal and external strategies specifically aimed at managing suicidal thoughts [35]—among those participants who used the app. Despite such promising initial evidence, it is important to note that the aforementioned existing apps are usually aimed at users of all age groups, without considering age-specific needs. Indeed, initial research on mental health apps highlights the importance of customization to enhance user experience and engagement among younger users [36,37]. Furthermore, these studies underscore the significance of providing clear, age-appropriate information, along with a simple yet engaging design tailored to the needs of younger users [36,37]. While adolescents and young adults have been involved in the development process of some safety planning apps [23,25], to the best of our knowledge, no safety planning smartphone app has specifically been developed for adolescents, and their effectiveness within this group remains unknown.

Furthermore, studies considering the duration of app use have shown that most participants used the respective safety planning app for no longer than 5 min during the entire study duration, primarily within the first two weeks after setup [34]. Porras-Segovia et al. [29] conducted a pilot study among adults to explore the acceptability of a safety planning smartphone app, where some participants reported being unable to remember the app during an emotional crisis. These findings highlight the importance of designing apps that are more seamlessly integrated into user’s daily life, thereby enhancing their accessibility in crisis situations. This could potentially be achieved by aligning development processes more closely with users’ specific needs and contextual realities.

In summary, existing research highlights the potential benefits of smartphone-based safety planning for suicide prevention. However, most existing studies have been conducted among adults and apps tailored to the specific needs of adolescents are rare. To address these gaps and contribute to suicide prevention among young people, we designed and developed a multi-component safety planning smartphone app to support adolescents in self-managing emotional crises. The aim of this paper is twofold: (1) to systematically explore the needs and preferences of key stakeholders (adolescents with lived experience, their parents, and practitioners) concerning smartphone-based safety planning, based on focus groups; and (2) to present the resulting structure and design of a newly developed multi-component safety planning smartphone app. The developed app integrates findings from the qualitative study as well as existing literature and pertinent theories, specifically tailored to support adolescents in the self-management of acute emotional crises.

## 2. Methods

### 2.1. Design

This study is part of the EMIRA project (ecological momentary intervention to reduce suicide risk among adolescents), which aims to develop and evaluate a safety planning smartphone app for adolescents. To ensure a user-centered structure and design, the app development was informed by qualitative focus groups with adolescents, practitioners, and parents, existing safety planning literature, as well as input from a community-based participatory research (CBPR) panel [38]. The gender-based CBPR panel consisted of three groups: (1) young adults with lived experience of suicidality, (2) practitioners—including psychologists and psychiatrists—with clinical expertise in adolescent mental health, and (3) researchers with expertise in suicide research from the fields of health sciences, social sciences, nursing science, and psychology. Ethical approval for the qualitative study was obtained from the Ethics Committee of Ulm University (393/22).

### 2.2. Participants and Recruitment

A convenience sample of adolescents aged 14–21 years was recruited from a regional inpatient child and adolescent psychiatric clinic via flyers and personal contact within the researchers’ network. Adolescents were eligible if they had experienced suicidal thoughts during the past 24 months and had prior experience with safety planning. Parents were included if they had children with suicidality and experience with safety planning, and were recruited by approaching them personally within the clinic and through the authors’ professional network. Practitioners working in adolescent psychiatry or psychotherapy were recruited through personal contact and a mailing list from the aforementioned psychiatric clinic. Written informed consent was required from all participants; for minors, consent from their legal guardians was mandatory. As an incentive, parents received €25 via bank transfer, while adolescents and practitioners received €25 shopping vouchers (wunschgutschein.de).

### 2.3. Phase 1: Qualitative Analysis

We conducted four focus groups (FG), each lasting approximately 90 min. These included two focus groups with seven female adolescents under the age of eighteen (FG 1: *n* = 3, FG 2: *n* = 4), one focus group with four mothers of children affected by suicidality (FG 3: *n* = 4), and one focus group with four female practitioners treating suicidal children and adolescents in inpatient or outpatient settings (FG 4: *n* = 4). The participants’ characteristics are presented in Table 1.

All focus groups were conducted in person at the clinic, except for the parent focus group, which was held online due to participants being located across different regions. Using a literature-based, pre-tested semi-structured format, the interview questions addressed participants’ experiences with safety planning and requirements for smartphone-based safety planning (see Appendix A, Appendix B and Appendix C). The questions were open ended, with specific prompts being used as needed. The same question outline was used for all participant groups, with adjustments made to reflect the specific perspectives and needs of each group. For adolescents, questions focused primarily on app structure and features, while for practitioners and parents, the emphasis was on general smartphone use and the handling of crisis situations. Focus groups were conducted by two female interviewers each—a PhD candidate and a researcher with lived experience—with no prior relationship to the participants before the commencement of the study (TG, JH, NB). Prior to the focus groups, the interviewers shared their personal and academic interest in the topic to enhance transparency. All focus groups were audio-recorded and transcribed verbatim.

The data coding process was conducted independently by two researchers—both PhD candidates with experiences in qualitative content analysis—using Kuckartz’s qualitative content analysis [39] within MAXQDA (version 6.10) (TG, JH). First, analytical units were defined. Next, relevant passages were paraphrased in simpler terms and generalized to a higher level of abstraction. The resulting condensed statements were then grouped into categories. In line with Kuckartz’s approach, the analysis combined deductive and inductive coding. This involves the identification of categories established by the interview guide (deductive) as well as categories emerging directly from the data (inductive). In a first step, broader main categories were identified, which were subsequently differentiated into more fine-grained subcategories. Through an iterative process of coding, reviewing, and refining, the category system was gradually refined and differentiated into main and subcategories. Discrepancies were discussed based on the descriptions of the categories in the category system until consensus was reached, and the procedure was repeated until no further relevant categories emerged. Following the analysis, we identified illustrative quotes and translated them from German to English, emphasizing the accurate reflection of their meaning rather than a verbatim translation.

### 2.4. Phase 2: App Development

After completing the qualitative content analysis, the app was designed and developed by a multiprofessional team with expertise in health sciences, social sciences, nursing sciences, psychology, information systems, and media design. App development was based on insights from the focus groups, existing literature, pertinent theories (e.g., dialectical-behavioral therapy (DBT), [40]), and feedback from a CBPR panel. These requirements were documented in user stories, i.e., brief descriptions of an app requirement from the users’ perspective [40]. The user stories were iteratively refined based on repeated feedback from adolescents and the CBPR panel to ensure ongoing involvement of the target group.

## 3. Results

The presentation of results is structured in two phases: Phase 1 focuses on the qualitative analysis (Section 3.1), and Phase 2 describes the app development (Section 3.2).

### 3.1. Phase 1: Qualitative Analysis

Participants provided general information on safety planning, as well as details about the app’s content, settings, and adjustability. Regarding the content of the app, participants talked about both app-based safety plans and additional features. All themes are presented in Table 2, with illustrative quotes for each theme. A detailed description of each theme and their implementation within the app is described below.

#### 3.1.1. General Information on Safety Planning

This category provides information on safety planning within mental health care in general, including safety plan development, storage, and evaluation. Practitioners described that, due to safety aspects, safety plans are typically created early in treatment, during the initial contact for outpatient care and after admission for inpatient care:

“*In therapeutic settings, the patient’s survival is considered the primary objective and is therefore usually addressed first*.”

*(Pr1)*

Interviewees reported that safety plans are usually created collaboratively by the affected individual and a practitioner, either individually or within a group. They also noted that parents or other social contacts are usually not involved in the development process, especially when the affected person is older than 18 years old. Additionally, once the plan is developed, social contacts are usually unaware of its content:

“*In the end, I’m the only one who more or less knows what’s on my safety plan*.”

*(A1)*

However, participants mentioned that the awareness of the safety plan depends—among other factors—on the adolescent’s ability to self-manage their emotional crisis. While individuals with chronic suicidal tendencies often felt less need to carry their safety plan with them all the time, those newly affected by suicidality considered it essential to permanently have their plan with them. Further, interviewees mentioned that safety plans typically include three (escalation) stages: self-distraction, involving social contacts, and seeking professional help. Participants stated that practitioners use guiding questions based on these stages, while considering the feasibility and availability of resources. Safety plans were often adapted to individual needs rather than including all standard components. Thereby, negative triggers (e.g., “call your friends!” when none exist) were to be avoided. The order of the content in the safety plan varied among adolescents, with only marginal changes and adjustments made, usually when the plan proved ineffective.

Overall, interviewees considered safety plans particularly useful in distressing or depressive situations:

“*The safety plan plays a big role for me, because I often find myself in situations where I’m not doing well. In those moments, I’m really glad to have something with me that can support me and help me to get through the situation*.”

*(A6)*

Despite such positive judgement, several reasons were noted for not using the safety plan (such as avoiding burdening others). In some cases, parents and practitioners observed that adolescents didn’t contact others during a crisis and were unsure why—an aspect that was explored further during interactions with professionals. Participants mentioned that the effectiveness of safety plans is often limited if they do not align with the specific situation:

“*Sometimes the [safety] plan doesn’t really help, for example when you are extremely angry.*”

*(A1)*

#### 3.1.2. Content of the App

##### Safety Plan

Regarding the app’s content, participants discussed the safety plan and its components, including warning signs, self-management, social distraction strategies, contact with trusted individuals and professionals, as well as the elimination of suicidal methods. They mentioned the usefulness of identifying warning signs: “*So maybe that it [the emotional crisis] doesn’t develop into something bigger, by paying attention to the early warning signs.*” *(A3)* Interviewees also talked about various measures for distraction (e.g., “*listening to music*” *(A1),* “*singing*” *(A2),* “*rubber bands, or these spiky balls that you can squeeze in your hand, hot chili candies, ice cubes, something like that*” *(Pa2)*) and their helpfulness in managing emotional distress. They also mentioned some distraction measures for which the app could offer support, including “*games*” *(A6)* or “*relaxation exercises*” *(A3).* Since multiple people (e.g., parents, friends) can be named as contacts on the safety plan, participants emphasized the importance of confirming the willingness of those listed as emergency contacts: “*Of course, I asked them whether it is okay for them to be contacted if I am in an emergency*” *(A3).* In private settings, contact to others typically involved in-person conversations, phone calls or text messages, often using messenger services (e.g., WhatsApp): “*I try to call my friends or text them so that I’m not alone and can distract myself from my thoughts*” *(A5).* Seeking professional help, such as contacting a practitioner, was identified as the most common way to access professional support. Interviewees suggested integrating a section in the smartphone-based safety plan for storing emergency numbers with direct links to contacts or chatrooms of crisis chat services, enabling communication with both (mental health) professionals and supportive peers with similar experiences. Regarding the ‘making the environment safer’ component, participants emphasized that removing dangerous objects should only occur with the affected person’s consent.

##### Additional App Features

Along with the app-based safety plan, participants mentioned additional app features. For instance, participants discussed the potential usefulness of mood barometers, using traffic light colors or emojis, to monitor current mood and track progress over time. They also suggested the possibility to save and view mood fluctuations graphically, including the option to share mood fluctuation graphs with others (e.g., via e-mail). Furthermore, participants emphasized the need for a function to record personal thoughts, either as text or audio, because “(…) *sometimes you don’t want to write*” *(A3)*. This would allow users, alongside the mood entry, to add additional information about why they are feeling a certain way at the moment. Additionally, participants mentioned the possibility of storing positive memories (texts, pictures, or videos) in the app:

“*I was thinking, like, when you’ve had a really good day, or seen something beautiful, like a sunset, it might be nice to save that in the app. Just as a way to store those moments, so when you’re feeling bad, you can remind yourself of the good things that have happened recently*.”

*(A3)*

#### 3.1.3. App Settings

The following statements address general app settings, focusing on privacy and data sharing. Participants highlighted the importance to store data only on the user’s personal device. Additionally, a snooze or deactivation function was recommended, allowing users to pause the app when needed. Furthermore, participants mentioned the importance of notifications in personalized intervals (e.g., mood tracking three times per day):

“*I think for a lot of people, including myself, it would be really nice if the app checked in with you in the morning, just asking how you’re doing. It’s a good way to become more aware of how you’re actually feeling, like: am I doing okay right now, or do I need some support?*”

*(A3)*

Partial access for others, such as therapists or parents, was seen as useful for collaborative reflection and progress tracking. However, it should be up to the individual user to decide whether and with whom they want to share app-based information. Interviewees recommended that the app name should be concise and discreet, ensuring its purpose is not immediately apparent to others.

#### 3.1.4. Adjustability

Within this category we group statements about user adjustability—an aspect that was highlighted by all interviewees—emphasizing the importance of individuality and variety in the app, such as the ability to personalize entries:

“*The ability to add new components, change or delete them, or even create entirely new ones. That kind of flexibility would be great. Just to have a bit more variety. Because if everything always stays exactly the same, I think, it gets boring for anyone*.”

*(A1)*

Regarding coping strategies, participants also highlighted the value of the possibility to try something new: “*not just* ‘*listen to your playlist*’, *but maybe at least try something new*” *(Pr4).* Therefore, various examples were provided, which could be randomly displayed as suggestions: “*yoga*” *(A1),* “*organize your drawer*” *(A3), or* “*learn five words in another language*” *(A3).*

### 3.2. Phase 2: App Development

**Figure 1:**
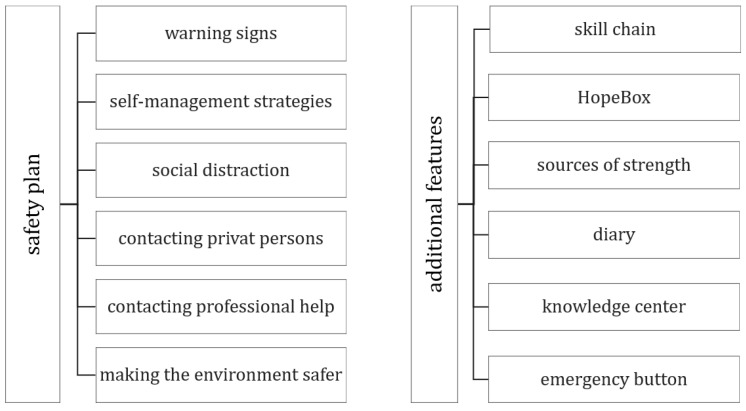
emira-app structure.

**Figure 2:**
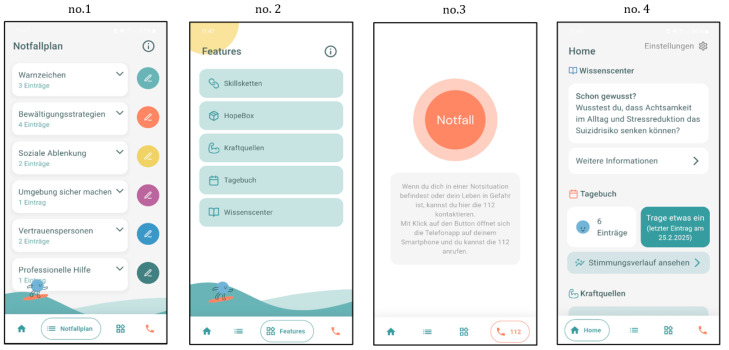
The panels show the safety plan (no. 1), the additional app features (no. 2), the emergency button (no. 3) and the homescreen (no. 4) of the emira-app.

The emira-app was designed to support adolescents in self-managing emotional crises; it is not limited to medical contexts, and is not tied to specific diagnoses. Its primary function is an app-based safety plan, supplemented by additional features to increase daily usage. The app structure is shown in Figure 1 and described below, while Figure 2 illustrates example screens from the app.

#### 3.2.1. General Information on Safety Planning

The emira-app was developed to allow users to personalize their entries in the safety plan. The entries can be made independently by the users or collaboratively, for example, with therapists. Within the app, users are informed about the potential benefits of communicating with others, such as a therapist, when developing the safety plan. Based on the focus group results, a safety plan is perceived as more supportive when its use is well explained, and the plan is carefully prepared. Therefore, the ‘knowledge center’ provides information about the safety planning concept, and the ‘homescreen’ encourages users to regularly review and assess their entries to ensure their effectiveness.

#### 3.2.2. Content

##### Safety Plan

The safety plan in the emira-app encompasses all components of the safety planning concept [cf. 9], which can be customized by sorting and editing. To recognize signs for an emotional crisis, users can record their warning signs. Further, for managing these crises, users can list their individual ‘internal coping strategies’ and strategies for ‘social distraction’, by using free text or by selecting from predefined suggestions, which were developed based on pertinent theories such as DBT. The suggestions are phrased in the first person to avoid technical jargon and align with the target group (e.g., “I cannot sleep”). To visualize warning signs and distraction measures across varying levels of distress, entries may be categorized into three levels: low, medium, and high. These levels can subsequently be color-coded—yellow, orange, and red, respectively—to indicate the severity of distress. The ‘try something new’ feature within ‘internal coping strategies’ provides additional suggestions for users to explore. If suggested strategies are perceived as helpful, they can be added to the personal list of strategies. To contact potential supporters (both private and professional), users can store relevant contacts either by importing them from their phone book or by adding them manually. Users can directly contact stored contacts, by calling or messaging them via WhatsApp or SMS. When choosing to contact someone via message, a pre-filled text (I’m not feeling well at the moment and need some help. Could you please contact me?) is displayed. This message can either be individually edited or replaced by a custom template text. Additionally, for professional help, suggested contacts are provided (e.g., U25, an email counseling service for adolescents). Finally, in ‘making the environment safer’, users can document individual agreements and measures for establishing a safer environment, either independently (e.g., avoiding high buildings) or with the help of others (e.g., having medications locked away).

##### Additional App Features

Furthermore, the app offers additional features designed to support self-management and self-efficacy: skill chains, HopeBox, sources of strength, diary, knowledge center, emergency button, and homescreen. As a distraction strategy based on DBT, ‘skill chains’ can be edited. These consist of multiple techniques or strategies that are practiced repeatedly in a fixed sequence to manage mental health symptoms effectively and can be used as a coping strategy within the app. Similar to the physical ‘Hope Kit’ [41], the emira-app provides a digital HopeBox, which can also be used as an individual coping strategy. The HopeBox represents a container of content, which serves as a reminder of positive life experiences and reasons for living, such as pictures of loved ones or inspirational quotes. Therefore, users can store their HopeBox content using various formats, such as texts, videos, and audios. To strengthen these positive associations, users can schedule notifications to be reminded of hopeful content stored in their HopeBox. Additionally, in ‘sources of strength’ users can add mindfulness activities, personal strengths, and gratitudes. As in the SPC ‘internal coping strategies’, within mindfulness activities, new ideas can be displayed and added if deemed useful. For those who enjoy journaling, the mood tracking function enables users to document their emotions using a slider with smiley faces and optional details like “What are you doing right now?”. Those entries can be displayed graphically to visualize the process, and filters are also available to identify factors that influence mood (e.g., being together with others), helping to pinpoint situations that have a positive or negative impact. Users can also add personal notes, either in text or audio. Similar to the HopeBox, users can set notifications, as reminders to reflect emotions in daily life. The ‘knowledge center’ offers easily understandable information on various topics, such as safety planning, suicidality, and stigma. Users get detailed information presented as well as short snippets designed to attract the interest in certain topics, like: “Did you know that childhood and adolescence are the phases of life in which suicidal tendencies often first occur?”. Upon opening the app, the ‘homescreen’ presents randomized entries from various app features (such as the safety plan and the HopeBox) and reminds users to make or check entries. Finally, an emergency button allows direct contact to the national emergency number (112), ensuring users receive help quickly whenever needed.

#### 3.2.3. App Settings

In the app settings users can activate a password function for privacy protection. Additionally, users can select which entries they wish to save and share (e.g., via PDF) and to whom (e.g., therapists, parents). All data is solely stored on the smartphone, with no exchange with external servers, enabling offline use without requiring an internet connection. Upon installation, an onboarding process guides users through the app setup and provides supplemental information about various app features. For privacy reasons, the app was named emira to avoid revealing its purpose.

#### 3.2.4. Adjustability

In the entire app, entries can be edited and sorted according to user preferences. Additionally, the components of the safety plan and the additional features can be sorted individually. Furthermore, users can set up their notifications individually, in the diary and in the HopeBox. They can also customize the color of the mascot ‘Emi’, that guides users through the app and is visually adapted in various ways to appeal to the target group.

## 4. Discussion

In this paper, we presented findings obtained from focus groups with key stakeholders (adolescents, parents, and practitioners) as well as described the structure and design of the safety planning smartphone app emira. To ensure that the emira-app meets the needs of all relevant key stakeholders, its development process was guided by close collaboration with adolescents, parents, and practitioners; continuous feedback from the target group; and a CBPR panel. The focus groups revealed a diverse array of needs and recommendations, encompassing details on safety planning in general as well as app features and technical requirements. Overall, no substantial differences were observed between participants’ perspectives; rather, their perspectives aligned and complemented one another. Consequently, a multi-component app was designed, incorporating both a smartphone-based safety plan as well as additional features, which may not only support adolescents in coping with emotional crises but also increase regular app usage. Additionally, the app’s design includes various customizable features, a visually appealing interface tailored to adolescents, and the incorporation of ‘Emi’ as an engaging mascot.

Like other safety planning apps (cf. [30]), the emira-app enables adolescents to create and store their safety plan digitally. Therefore, the emira safety plan incorporates all six SPCs, including ‘making the environment safer’, which was not implemented within all available safety planning apps (cf. [30]), but plays a critical role in reducing suicide risk [9]. Based on participant recommendations, users can personalize the app and its entries according to their individual preferences: Entries can be sorted individually, and new distraction strategies can be tried and added to the safety plan. Additionally, based on the results of the focus groups, users can directly contact those contacts listed in their stored safety plan and choose between different communication methods (e.g., call or message). Another key adaptation is the inclusion of predefined suggestions: Users of the emira-app can enter information into the SPCs as free text, but also select them from a list of predefined suggestions. The suggested entries in the SPC ‘warning signs’ and ‘coping strategies’ align with the most frequently reported entries, as identified by Porras-Segovia et al. [29].

In addition to the safety plan, the emira-app offers additional app features. For example, as recommended by participants, a diary feature was introduced, allowing users to add entries either via text or audio and view their mood entries graphically. This feature is similar to the one used in the Strength Within Me app [27], where users can document their daily experiences. This aligns with research emphasizing the importance of integrating additional features beyond the core safety plan [25]. Accordingly, the emira-app, like the BeyondNow app [25], features an emergency button to call the German emergency number, ensuring that users receive help whenever needed. Additionally, as pointed out by participants in the focus groups, a digital HopeBox was implemented. Consistent with recommendations from other studies [42], the emira-app allows users to store content in the HopeBox in different formats. As highlighted by adolescents in the focus groups, notifications were implemented with the possibility to customize them individually. This aligns with other app evaluations that emphasize the importance of reminders to encourage consistent use, as demonstrated by the MEmind app [29]. To address this, the emira-app enhances personalization by allowing users to set individual notifications, facilitating sustained engagement with the app.

Additionally, the emira-app offers various customization options (e.g., adjusting the color of the mascot, Emi), while the integrated ‘homescreen’ and ‘try something new’ features ensure an interactive and engaging experience. These elements enhance flexibility and personalization, making the app attractive to a broader target group while encouraging app use beyond crisis situations and integration into daily life. To address confidentiality, user data is only stored on the local device, and similar to the SafetyPlan-app [15], password protection can be activated, allowing users to control access and determine which app content to share and with whom. This approach aligns with data security measures implemented in other safety planning smartphone apps [23,24,32].

Given the importance of a clear, user-friendly handling [25,43] and intuitive use [15], we emphasized a simple user interface. Nonetheless, the emira-app includes an in-app tutorial and supplementary information to ensure easy handling. Regarding the design of the safety plan, the app-based format closely replicates the structure of conventional paper-based plans to facilitate a smooth transition for users already familiar with the latter.

Beyond these design aspects, it is important to situate the emira-app within the broader context of smartphone use and its implications for mental health among adolescents. The emira-app enables adolescents to access their safety plan during emotional crisis and thereby aims to support their coping in such situations; however, not all young people have equal access to digital technologies, nor should the responsibility for managing mental health crises rest solely on individuals [44]. The safety planning concept on which the app is based also encompasses additional sources of support, including both private and professional opportunities for help. Accordingly, the app provides information about other sources of support, such as trusted persons, crisis helplines, and professional services. Additionally, previous research highlights the central but ambiguous role of smartphones in daily life among adults (e.g., [45,46]). Extending this perspective to younger populations, mental health apps for adolescents, such as the emira-app, require discussion of the potential benefits and possible adverse side effects on youth mental health. While some studies among adolescents report positive outcomes, including short-term mood improvements [47], others link smartphone use among this group to reduced quality of life [48] and psychiatric disorders [49]. At the same time, smartphone apps are increasingly seen as a potential way to provide access to mental health support and evidence-based care (e.g., [50]). This highlights the importance to evaluate the developed emira-app in a randomized controlled trial (RCT), focusing on both beneficial outcomes as well as unwanted side effects.

### Strengths and Limitations

Among adolescents, the focus group sample consisted only of female participants, which can be explained by the higher likelihood of girls seeking help compared to boys, particularly during adolescence [51,52,53], making them more prevalent in inpatient settings. Additionally, the sample size was predetermined based on information power and feasibility, reflecting the study’s focus on practical insights. Therefore, the findings cannot be generalized, as they are based on a qualitative study with a limited and homogenous sample. This limitation is highlighted by heterogenous evidence on gender-related smartphone use, with some studies indicating higher and distinct use among female adolescents [48,54,55], while others report mixed or inconsistent evidence [56,57]. In particular, the exclusive participation of German female adolescents highlights the need for future intersectional studies to adopt a broader perspective on adolescents’ experiences of suicidality, safety planning and their engagement with smartphone apps. Moreover, the empirical evaluation of the developed app is still pending, underscoring the need for an evaluation study to provide evidence on the app’s effectiveness and user behavior.

Despite these limitations, the emira-app aims to address the needs for smartphone-based safety planning, supporting adolescents in self-managing emotional crises. The app development followed a participatory approach engaging adolescents with lived experience, their parents, and practitioners. Further, additional features have been integrated to encourage the app’s use in everyday life, facilitating its effectiveness during crisis situations. The app’s compatibility with both, iPhone and Android, further enhances its accessibility. Although initially developed in Germany, after its evaluation the app may be adapted for other countries with minor adjustments, such as updating the crisis helpline information. While the potential of the app is promising, potential unwanted side effects—such as adolescents experiencing strain due to high screen time—cannot be ruled out and should be examined through scientific evaluation prior to implementation.

## 5. Conclusions

The emira-app is a safety planning smartphone app specifically designed for adolescents to support their self-management during emotional crises and thereby make a contribution to suicide prevention. Its development was informed by the involvement of key stakeholders, including adolescents, parents, and practitioners. Overall, the ability for users to customize and configure the app individually appears to be significant and was consistently taken into account during the app’s development. To evaluate the effectiveness of the emira-app in enhancing suicide-related coping among adolescents with mental health issues, an RCT is currently being conducted.

## Figures and Tables

**Table 1:** Participant characteristics.

Adolescents				
	age (years)	sex	ICD-10 diagnosis	setting
A1	17	female	F3X	inpatient
A2	16	female	F3X	inpatient
A3	15	female	F5X	inpatient
A4	13	female	unknown	inpatient
A5	17	female	F6X	inpatient
A6	16	female	F3X	inpatient
A7	16	female	F6X	inpatient
**Practitioners**				
	age (years)	sex	education	setting
Pr1	43	female	psychiatrist	outpatient
Pr2	30	female	psychologist	inpatient
Pr3	36	female	nurse	inpatient
Pr4	37	female	psychiatrist	outpatient
**Parents**				
	age (years)	sex	child died by suicide	child with SI during recruitment
Pa1	52	female	yes	no
Pa2	54	female	no	yes
Pa3	57	female	yes	yes
Pa4	50	female	no	yes

Note: F3X = mood [affective] disorders, F5X = behavioural syndromes associated with physiological disturbances and physical factors, F6X = disorders of adult personality and behaviour.

**Table 2:** Themes and illustrative quotes for each theme (translated by the authors).

Theme	Subtheme	Examples
General information on safety planning		“I’ve got a few things listed on it [my safety plan], and I keep adding things to it whenever I realize something else has helped me.” (A3) “If I know I’m working with a patient who might need something like this [a safety plan], I try to walk through a scenario with them, one where they’d actually have to use their safety plan. Then we think through the steps they could take to reduce the risk of anything serious happening. We start with the low-threshold options, the immediate surroundings, like: is there someone you can turn to? Or even first, what can you do to calm yourself down? Then we move on to involving other people, step by step, and if none of that works, we go all the way to the more serious actions, like calling the ambulance or the clinic.” (Pr1)
Content of the app	Safety plan	“Just a suggestion [in the app] for something to help with distraction. Ideally, a tool offering a variety of options. Perhaps with the ability to add personal favorites that could then be integrated into the safety plan.” (A3)
	Additional app features	“ (…) I also have to do this on the [clinic] ward: every evening I have to write down three positive things from the day.” (A5)
App settings		“For example, receiving messages that pop up, such as ‘You’re amazing’.” (A6)
Adjustability		“Well, if we’re in a good mood, it might be helpful to have the option to turn this feature off. Or at least, maybe having a button to disable notifications. (…) Because if it’s always on, it could potentially become annoying after a while (…) but that probably depends on the individual.” (A1)

## Data Availability

Due to ethical and legal constraints, the interview transcripts cannot be shared. Participants did not provide consent for data sharing beyond the scope of this study.

## Abbreviations

app	application
CBPR	community-based participatory research
DBT	dialectical behavioral therapy
EMIRA	ecological momentary intervention to reduce suicide risk among adolescents
FG	focus group
SI	suicidal ideation
SPC	safety plan components
RCT	randomized controlled trial

## Appendix A

**Table A1:** Interview guide for adolescents.

Theme 1: Dealing with crises	
(1) All of you experienced crises, i.e., situations in which you were not feeling good.	
Identify helpful resources in crisis situations	What helps you when you are not feeling good? ○ Who do you turn to when you are not feeling good?
**Theme 2: Experiences with safety planning**	
(2) I am sure you have all talked to your therapist about measures that can help you in a crisis situation. This often results in a whole list of things.	
Safety planning processes Identify people involved	How does the development of such a list work? ○ How were you involved in the development of your safety plan? ○ In which situation was it created? ○ Who was involved in the development of your safety plan? (ask if necessary: parents, family, friends?) ○ Who, apart from you, knows your safety plan? (If necessary, ask: What do your parents/friends/teachers know about it?)
(3) The purpose of a safety plan is to support you in a crisis.	
Positive/negative experiences with safety planning	What role does your safety plan play in your everyday life? In which situation has your safety plan ever been helpful? ○ What exactly was helpful? ○ What do you like about your safety plan? In which situation could your safety plan not support you as you had hoped? ○ Why was that the case? ○ What do you think is not good about your safety plan?
**Theme 3: Safety planning app**	
(4) Nowadays, there is hardly anyone who doesn’t have a smartphone. We use it to buy a bus ticket, to stay in contact with our friends, to create a shopping list and much more.	
Cell phone use/app use General	Which apps do you like to use? What do you like about these apps? ○ How do you decide for or against using an app? In which situations do you not care about your smartphone? In which situations is your smartphone particularly important to you? What role does your smartphone play when you are not feeling good?
(5) There are now numerous apps that can help with mental stress.	
Use of MH apps	What experiences do you have with apps that can help you in a crisis situation? ○ Which apps do you know? ○ Which apps do you particularly like?
(6) You already know that we want to develop an app that allows you to save and manage your safety plan on your smartphone.	
Evaluation of app-based safety planning	What should such an app be able to do/ What would you want from such an app? What would be your concerns?
(7) I would now ask you to write down specifically which functions you would like to see in our app. Please take a new piece of paper for each function.	
Identify and evaluate functions	Collect the functions together; group them on the flipchart Complete with your own prepared cards ○ Recognize warning signs ○ Internal coping strategies—things I can do to distract my mind from problems without contacting another person ○ Social contacts that can distract you ○ Family members or friends who can help you ○ Contact points that can help you ○ Making your environment safe ○ Emergency button ○ … ○ … I will now give you all 6 sticky dots. Please stick three/two/one dot on the component that you like best/second best/third best. Discuss together: Agreement, surprise

## Appendix B

**Table A2:** Interview guide for parents.

Theme 1: Crises of your child	
(1) Suicidal thoughts are unfortunately a common phenomenon among adolescents. All of you are parents of a child who has been in such a crisis situation.	
Identify helpful resources in crisis situations	In which situations is your child feeling good? ○ What makes your child happy/satisfied? What helps your child when he/she is not feeling good? ○ How can you support your child when he/she is not feeling good—In which situations is your child feeling good? What makes your child happy/satisfied? What helps your child when he/she is not feeling good? ○ How can you support your child when he/she is not doing well?
**Theme 2: Experiences with safety planning**	
(2) Drawing up a safety plan is an important component in the treatment of adolescents at risk of suicide. Such a safety plan contains measures that, when applied in a crisis situation, can reduce the risk of suicidal behavior.	
Safety planning processes Identify people involved	What do you know about the development of such safety plans? ○ What is/was your role as a parent in the development of the safety plan? In which situations does your child use his/her safety plan? ○ What is your role as a parent in the application of the safety plan? What is the content of your child’s safety plan?
(3) Safety plans should be used at the latest when adolescents find themselves in an emotional crisis situation and have suicidal thoughts.	
Positive/negative experiences with safety planning. Application barriers	Can you think of any situations in which the safety plan was helpful for your child? ○ What exactly was helpful? Can you think of situations in which the safety plan was not able to provide the support you had hoped for? ○ Why was this the case?
**Theme 3: Safety planning app**	
(4) Nowadays, there is hardly anyone who doesn’t have a smartphone. Adolescents in particular are said to use their smartphone intensively.	
Smartphone use of the child	What role does the smartphone play in your child’s life? ○ In which situations does your child use his/her smartphone particularly often? ○ In which situations does your child hardly use his/her smartphone at all? How does your child use his/her smartphone when he/she is not feeling good?
(5) There are now also numerous apps that can provide support in dealing with mental stress.	
Use of MH apps	Do you know such apps? Does your child use such apps? ○ Which ones exactly? What kind of apps do you find useful for adolescents to support them in mentally stressful situations? ○ What exactly do you find useful?
(6) As you already know, we would like to develop an app in which adolescents can save and manage their safety plan.	
Evaluation of app-based safety planning	How do you feel about the idea of your child using such an app? ○ What concerns would you have? ○ What advantages would such an app bring? ○ What functions would you like to see in the app? To what extent would you as a parent like to be involved in the use of the app?

## Appendix C

**Table A3:** Interview guide for practitioners.

Theme 1: Experiences safety planning	
(1) They all have experience in treating adolescents who experience suicidal tendencies. Drawing up a safety plan is an important component of the treatment of adolescents at risk of suicide.	
Safety planning processes Identify people involved	How does the creation of such a safety plan work? ○ Which people are involved in drawing up the plan? (if necessary, ask: parents, other caregivers) ○ What roles do you have in the preparation? ○ What role do adolescents have in drawing up the plan? ○ When does the creation take place? How is the safety plan kept by adolescents? When is the safety plan used? ○ Do adolescents report on the use of the safety plan? Under what circumstances is the safety plan revised?
(2) Safety plans should be used at the latest when adolescents are in an emotional crisis situation and have suicidal thoughts.	
Positive/negative experiences with safety planning; Application barriers	How do you rate the effectiveness of safety plans? ○ And why? What feedback do adolescents give regarding the usefulness of their safety plans? ○ Can you think of any situations in which a safety plan was helpful for an adolescent? What exactly was helpful for an adolescent? ○ Can you think of situations in which a safety plan was not able to provide the support you had hoped for? Why was this the case?
(3) Even if there are typical components of a safety plan, every safety plan is very personal and can be very individually designed.	
	How do you choose which components to include in the safety plan? ○ Are there any components that you prefer and why? ○ Are there any components that you do not like to use and why? How do you choose which components to include in the safety plan? ○ Are there any components that you prefer and why? ○ Are there any components that you do not like to use and why?
**Theme 2: Safety planning app**	
(4) Nowadays, there is hardly anyone who doesn’t have a smartphone. We use it to buy a bus ticket, to stay in contact with our friends, to create a shopping list and much more. There are now also numerous apps that can help us deal with mental stress. They are also known as mental health apps.	
Use of MH apps	Do you know such apps? Have you ever recommended certain mental health apps to adolescents? ○ Which ones were they? ○ Why do you find them useful?
(5) As you already know, we would like to develop an app in which adolescents can save and manage their safety plan.	
Evaluation of app-based safety planning	Under what circumstances would you use such an app in treatment? ○ Which functions would you like to see? ○ What concerns would you have? ○ What advantages would such an app bring?
(6) If possible, we would like to design the app so that it cannot only be used when needed, but also actively asks adolescents at regular intervals during the day how they are doing and reminds them of their safety plan.	
Evaluation of active app	What do you think of this idea? ○ What concerns do you have? ○ What advantages would this bring?

## References

[1] World Health Organization (2025). Suicide Worldwide in 2021: Global Health Estimates.

[2] Evans E., Hawton K., Rodham K., Deeks J. (2005). The prevalence of suicidal phenomena in adolescents: A systematic review of population-based studies. Suicide Life Threat. Behav..

[3] Alqueza K.L., Pagliaccio D., Durham K., Srinivasan A., Stewart J.G., Auerbach R.P. (2023). Suicidal Thoughts and Behaviors Among Adolescent Psychiatric Inpatients. Arch. Suicide Res..

[4] Hawton K., Saunders K.E.A., O’Connor R.C. (2012). Self-harm and suicide in adolescents. Lancet.

[5] Destatis (2024). Todesursachen Suizide.

[6] Plener P.L., Kaess M., Fegert J., Resch F., Plener P. (2023). Suizidalität im Kindes- und Jugendalter. Psychiatrie und Psychotherapie des Kindes- und Jugendalters.

[7] Gili M., Castellví P., Vives M., de la Torre-Luque A., Almenara J., Blasco M.J., Cebrià A.I., Gabilondo A., Pérez-Ara M.A., A M.-M. (2019). Mental disorders as risk factors for suicidal behavior in young people: A meta-analysis and systematic review of longitudinal studies. J. Affect. Disord..

[8] Voss C., Ollmann T.M., Miché M., Venz J., Hoyer J., Pieper L., Höfler M., Beesdo-Baum K. (2019). Prevalence, Onset, and Course of Suicidal Behavior Among Adolescents and Young Adults in Germany. JAMA Netw. Open.

[9] Stanley B., Brown G.K. (2012). Safety Planning Intervention: A Brief Intervention to Mitigate Suicide Risk. Cogn. Behav. Pract..

[10] Stanley B., Brown G.K., Brenner L.A., Galfalvy H.C., Currier G.W., Knox K.L., Chaudhury S.R., Bush A.L., Green K.L. (2018). Comparison of the Safety Planning Intervention with Follow-up vs Usual Care of Suicidal Patients Treated in the Emergency Department. JAMA Psychiatry.

[11] Nuij C., van Ballegooijen W., de Beurs D., Juniar D., Erlangsen A., Portzky G., O’COnnor R.C., Smit J.H., Kerkhof A., Riper H. (2021). Safety planning-type interventions for suicide prevention: Meta-analysis. Br. J. Psychiatry.

[12] Ferguson M., Rhodes K., Loughhead M., McIntyre H., Procter N. (2022). The Effectiveness of the Safety Planning Intervention for Adults Experiencing Suicide-Related Distress: A Systematic Review. Arch. Suicide Res..

[13] Abbott-Smith S., Ring N., Dougall N., Davey J. (2023). Suicide prevention: What does the evidence show for the effectiveness of safety planning for children and young people?—A systematic scoping review. J. Psychiatr. Ment. Health Nurs..

[14] May A.M., Al-Dajani N., Ballard E.D., Czyz E. (2023). Safety plan use in the daily lives of adolescents after psychiatric hospitalization. Suicide Life Threat. Behav..

[15] O’Grady C., Melia R., Bogue J., O’Sullivan M., Young K., Duggan J. (2020). A Mobile Health Approach for Improving Outcomes in Suicide Prevention (SafePlan). J. Med. Internet Res..

[16] Kennard B.D., Biernesser C., Wolfe K.L., Foxwell A.A., Lee S.J.C., Rial K.V., Patel S., Cheng C., Goldstein T., McMakin D. (2015). Developing a brief suicide prevention intervention and mobile phone application: A qualitative report. J. Technol. Hum. Serv..

[17] Medienpädagogischer Forschungsverbund Südwest (2022). JIM 2022: Basisuntersuchung zum Medienumgang 12- bis 19-Jähriger in Deutschland.

[18] Gregory J.M., Sukhera J., Taylor-Gates M. (2017). Integrating smartphone technology at the time of discharge from a child and adolescent inpatient psychiatry unit. J. Can. Acad. Child. Adolesc. Psychiatry.

[19] Myin-Germeys I., Klippel A., Steinhart H., Reininghaus U. (2016). Ecological momentary interventions in psychiatry. Curr. Opin. Psychiatry.

[20] Bush N.E., Dobscha S.K., Crumpton R., Denneson L.M., Hoffman J.E., Crain A., Cromer R., Kinn J.T. (2015). A Virtual Hope Box smartphone app as an accessory to therapy: Proof-of-concept in a clinical sample of veterans. Suicide Life Threat. Behav..

[21] Denneson L.M., Smolenski D.J., Bauer B.W., Dobscha S.K., Bush N.E. (2019). The Mediating Role of Coping Self-Efficacy in Hope Box Use and Suicidal Ideation Severity. Arch. Suicide Res..

[22] Dubad M., Elahi F., Marwaha S. (2021). The Clinical Impacts of Mobile Mood-Monitoring in Young People with Mental Health Problems: The MeMO Study. Front. Psychiatry.

[23] Pauwels K., Aerts S., Muijzers E., Jaegere Ede van Heeringen K., Portzky G. (2017). BackUp: Development and evaluation of a smart-phone application for coping with suicidal crises. PLoS ONE.

[24] Skovgaard Larsen J.L., Frandsen H., Erlangsen A. (2016). MYPLAN—A Mobile Phone Application for Supporting People at Risk of Suicide. Crisis.

[25] Melvin G.A., Gresham D., Beaton S., Coles J., Tonge B.J., Gordon M.S., Stanley B. (2019). Evaluating the feasibility and effectiveness of an Australian safety planning smartphone application: A pilot study within a tertiary mental health service. Suicide Life Threat. Behav..

[26] O’Toole M.S., Arendt M.B., Pedersen C.M. (2019). Testing an App-Assisted Treatment for Suicide Prevention in a Randomized Controlled Trial: Effects on Suicide Risk and Depression. Behav. Ther..

[27] Bruen A.J., Wall A., Haines-Delmont A., Perkins E. (2020). Exploring Suicidal Ideation Using an Innovative Mobile App-Strength Within Me: The Usability and Acceptability of Setting up a Trial Involving Mobile Technology and Mental Health Service Users. JMIR Ment. Health.

[28] Jeong Y.W., Chang H.J., Kim J.A. (2020). Development and Feasibility of a Safety Plan Mobile Application for Adolescent Suicide Attempt Survivors. Comput. Inform. Nurs..

[29] Porras-Segovia A., De Granda-Beltrán A.M., Gallardo C., Abascal-Peiró S., Barrigón M.L., Artés-Rodríguez A., López-Castroman J., Courtet P., Baca-García E. (2024). Smartphone-based safety plan for suicidal crisis: The SmartCrisis 2.0 pilot study. J. Psychiatr. Res..

[30] Gryglewicz K., Orr V.L., McNeil M.J., Taliaferro L.A., Hines S., Duffy T.L., Wisniewski P.J. (2024). Translating Suicide Safety Planning Components into the Design of mHealth App Features: Systematic Review. JMIR Ment. Health.

[31] Buus N., Juel A., Haskelberg H., Frandsen H., Larsen J.L.S., River J., Andreasson K., Nordentoft M., Davenport T., Erlangsen A. (2019). User Involvement in Developing the MYPLAN Mobile Phone Safety Plan App for People in Suicidal Crisis: Case Study. JMIR Ment. Health.

[32] Meier L., Gurtner C., Nuessli S., Miletic M., Bürkle T., Durrer M. (2022). SERO—A New Mobile App for Suicide Prevention. Stud Health Technol. Inform..

[33] Buus N., Erlangsen A., River J., Andreasson K., Frandsen H., Larsen J.L.S., Nordentoft M., Juel A. (2020). Stakeholder Perspectives on Using and Developing the MYPLAN Suicide Prevention Mobile Phone Application: A Focus Group Study. Arch. Suicide Res..

[34] Rainbow C., Tatnell R., Blashki G., Fuller-Tyszkiewicz M., Melvin G.A. (2024). Digital safety plan effectiveness and use: Findings from a three-month longitudinal study. Psychiatry Res..

[35] Stanley B., Green K.L., Ghahramanlou-Holloway M., Brenner L.A., Brown G.K. (2017). The construct and measurement of suicide-related coping. Psychiatry Res..

[36] Garrido S., Cheers D., Boydell K., Nguyen Q.V., Schubert E., Dunne L., Meade T. (2019). Young People’s Response to Six Smartphone Apps for Anxiety and Depression: Focus Group Study. JMIR Ment. Health.

[37] Høgsdal H., Kyrrestad H., Rye M., Kaiser S. (2024). Exploring Adolescents’ Attitudes Toward Mental Health Apps: Concurrent Mixed Methods Study. JMIR Form. Res..

[38] Wallerstein N., Duran B., Oetzel J.G., Minkler M. (2018). Community-Based Participatory Research for Health: Advancing Social and Health Equity.

[39] Kuckartz U., Rädiker S. (2023). Qualitative Content Analysis: Methods, Practice and Software.

[40] Cohn M. (2004). User Stories Applied: For Agile Software Development.

[41] Wenzel A., Brown G.K., Beck A.T. (2009). Cognitive Therapy for Suicidal Patients: Scientific and Clinical Applications.

[42] Le Jeannic A., Turmaine K., Gandré C., Vinet M.-A., Michel M., Chevreul K. (2023). Defining the Characteristics of an e-Health Tool for Suicide Primary Prevention in the General Population: The StopBlues Case in France. Int. J. Environ. Res. Public Health.

[43] Nuij C., van Ballegooijen W., de Beurs D., de Winter R.F.P., Gilissen R., O’cOnnor R.C., Smit J.H., Kerkhof A., Riper H. (2022). The feasibility of using smartphone apps as treatment components for depressed suicidal outpatients. Front. Psychiatry.

[44] Lupton D. (2013). The digitally engaged patient: Self-monitoring and self-care in the digital health era. Soc. Theory Health.

[45] Hawkins C., Awondo P., Miller D. (2024). An Anthropological Approach to mHealth.

[46] Duque M. (2022). Ageing with Smartphones in Urban Brazil: A Work in Progress.

[47] Minich M., Moreno M. (2024). Real-world adolescent smartphone use is associated with improvements in mood: An ecological momentary assessment study. PLoS ONE.

[48] Poulain T., Meigen C., Kiess W., Vogel M. (2025). Smartphone use, wellbeing, and their association in children. Pediatr. Res..

[49] Wacks Y., Weinstein A.M. (2021). Excessive Smartphone Use Is Associated With Health Problems in Adolescents and Young Adults. Front. Psychiatry.

[50] Price M., Yuen E.K., Goetter E.M., Herbert J.D., Forman E.M., Acierno R., Ruggiero K.J. (2014). mHealth: A mechanism to deliver more accessible, more effective mental health care. Clin. Psychol. Psychother..

[51] Weiss M., Hildebrand A., Stemmler M. (2024). Inanspruchnahme psychosozialer Hilfen bei jungen Erwachsenen mit suizidalem Erleben und Verhalten. Psychother. Psychosom. Med. Psychol..

[52] Schwake C. (2022). Akut-Inanspruchnahme von Kinder- und Jugendpsychiatrischer Behandlung und ihre Veränderung in einem Zeitraum von fünf Jahren (2011–2015): Eine Retrospektive Querschnittsstudie in Einem Multizentrischen Deutschen Kinder- und Jugendpsychiatrischen Patientenkollektiv.

[53] Plener P.L., Groschwitz R.C., Franke C., Fegert J.M., Freyberger H.J. (2015). Die stationäre psychiatrische Versorgung Adoleszenter in Deutschland. Z. Für Psychiatr. Psychol. Und Psychother..

[54] Sánchez-Martínez M., Otero A. (2009). Factors associated with cell phone use in adolescents in the community of Madrid (Spain). Cyberpsychol. Behav..

[55] Medienpädagogischer Forschungsverbund Südwest (2024). JIM-Studie 2024: Basisuntersuchung zum Medienumgang 12- bis 19-Jähriger: Mpfs.

[56] Lopez-Fernandes O., Riva G., Wiederhold B.K., Cipresso P. (2015). Problem mobile phone use in Spanish and British adolescents: First steps towards a cross-cultural research in Europe. The Psychology of Social Networking.

[57] Soni R., Upadhyay R., Jain M. (2017). Prevalence of smart phone addiction, sleep quality and associated behaviour problems in adolescents. Int. J. Res. Med. Sci..

